# The spectrum of electrolyte abnormalities in black African people living with human immunodeficiency virus and diabetes mellitus at Edendale Hospital, Pietermaritzburg, South Africa

**DOI:** 10.4102/sajhivmed.v21i1.1095

**Published:** 2020-07-23

**Authors:** Preyanka Pillay, Somasundram Pillay, Nobuhle Mchunu

**Affiliations:** 1 Department of Internal Medicine, Greys Hospital, Pietermaritzburg, South Africa; 2 School of Clinical Medicine, College of Health Sciences, University of KwaZulu-Natal, Durban, South Africa; 3 Department of Internal Medicine, Edendale Hospital, Pietermaritzburg, South Africa; 4 Department of Internal Medicine, King Edward Hospital, Durban, South Africa; 5 Department of Biostatistics, Faculty of Statistics, South African Medical Research Council, Durban, South Africa; 6 Department of Statistics, School of Mathematics, Statistics and Computer Science, University of KwaZulu-Natal, Pietermaritzburg, South Africa

**Keywords:** HIV, diabetes mellitus, electrolytes, sodium, potassium, calcium, phosphate, black African

## Abstract

**Background:**

Serum electrolyte abnormalities in black African people living with human immunodeficiency virus (HIV) and diabetes mellitus (PLWH/DM) is unknown.

**Objectives:**

The aim of this study was to analyse serum electrolytes (sodium, potassium, calcium and phosphate) and factors associated with electrolyte abnormalities in black African PLWH/DM versus HIV-uninfected patients with DM.

**Methods:**

We conducted a retrospective case-control study in 96 black African PLWH/DM (cases) and 192 HIV-uninfected patients with DM (controls), who were visiting the Edendale Hospital DM clinic, from 01 January 2016 to 31 December 2016. Pearson’s correlation, multivariate linear and logistic regression analyses were utilised.

**Results:**

Hypocalcaemia was the most frequent electrolyte abnormality in PLWH/DM and HIV-uninfected patients with DM (31.25% vs. 22.91%), followed by hyponatraemia (18.75% vs. 13.54%). Median (IQR) corrected serum calcium levels were significantly lower in PLWH/DM compared with HIV-uninfected patients with DM (2.24 [2.18–2.30] mmol/L vs. 2.29 [2.20–2.36] mmol/L; *p* = 0.001). For every per cent increase in glycated haemoglobin, the odds of hyponatraemia significantly increased in both PLWH/DM (odds ratio [OR]: 1.55; 95% confidence interval [CI]: 1.19 –2.02; *p* = 0.003) and HIV-uninfected patients with DM (OR: 1.26; 95% CI: 1.04 –1.54; *p* = 0.009).

**Conclusion:**

Hypocalcaemia and hyponatraemia were the most frequent electrolyte abnormalities and occurred more frequently in PLWH/DM compared with HIV-uninfected patients with DM. People living with HIV and DM have significantly lower corrected serum calcium levels compared with HIV-uninfected patients with DM. Furthermore, hyponatraemia is a marker of impaired glycaemic control.

## Introduction

Low- and middle-income countries account for 80% of the global diabetes mellitus (DM) burden.^1^ The International Diabetes Federation (IDF) estimates that Africa will experience the greatest global upsurge of DM by 2045.^1^ In 2015, DM was the second leading cause of mortality in South Africa after tuberculosis.^2^ Moreover, South Africa has the highest prevalence of human immunodeficiency virus (HIV) globally and recorded 7.06 million people living with HIV (PLWH) in 2017, with KwaZulu-Natal province having the majority of these cases.^3,4^ Following the introduction of antiretroviral therapy (ART), the prevalence of comorbid HIV and DM is on the rise because of an increase in life expectancy and the adverse metabolic effects of ART.^5^ A study in the United States of America (USA) determined that the prevalence of DM in PLWH was 10.3%, and the prevalence of DM was 3.8% higher in PLWH compared with the general population.^6^

Electrolytes play a vital role in maintaining homeostasis and are paramount in mediating enzymatic reactions, cellular function and electrical gradients.^7^ Patients with HIV or DM are predisposed to electrolyte abnormalities because of multifactorial pathophysiological factors.^8,9^ The risk of nephropathy, with subsequent electrolyte abnormalities, increases in the setting of comorbid HIV and DM. Furthermore, the black African population has distinct electrolyte physiology and a predisposition to chronic kidney disease and HIV-associated nephropathy (HIVAN).^10^ In addition, the use of tenofovir (TDF) increases the risk of proximal tubular dysfunction and subsequent hypokalaemia and hypophosphataemia.^11^

The Atherosclerosis Risk in Communities (ARIC) study concluded that African American patients had an approximately twofold greater incidence of type 2 DM compared with white patients.^12^ Although this racial disparity is multifactorial, lower vitamin D^13^ and serum potassium levels^14^ in the black population are being explored as possible contributory factors. Importantly, the prevalence of vitamin D deficiency in type 2 DM is disproportionately elevated in African American people compared with other ethnic groups in the USA.^15^

Electrolyte abnormalities are associated with increased morbidity and mortality, even if they are chronic or of mild severity and may remain clinically silent until an advanced stage.^8,9^ However, there is a paucity of data from Africa regarding electrolyte abnormalities in HIV or DM. Furthermore, there are no studies assessing electrolyte abnormalities in black African people living with HIV and diabetes mellitus (PLWH/DM). Determining and understanding the spectrum of electrolyte abnormalities in black African PLWH/DM are of crucial significance, particularly in South Africa, which has a large burden of HIV and DM and is undergoing an epidemiological transition in a resource-limited setting.

The objective of this retrospective case–control study was to determine, compare and identify associated factors regarding serum electrolyte abnormalities (sodium, potassium, calcium and phosphate) in black African PLWH/DM versus black African HIV-uninfected patients with DM who attended the Edendale Hospital DM clinic from 01 January to 31 December 2016.

## Methods

This quantitative retrospective case–control study was conducted in 96 black African PLWH/DM (cases) and 192 black African HIV-uninfected patients with DM (controls) attending the Edendale Hospital DM clinic, Pietermaritzburg, KwaZulu-Natal, South Africa, over 1 year from 01 January to 31 December 2016. Records of patients attending the DM clinic were analysed retrospectively from datasheets. Electrolytes were measured during routine outpatient visits at the Edendale Hospital DM clinic. Black African PLWH/DM included in the study could have either type 1 or type 2 DM, be on ART or antiretroviral-naïve, have any degree of renal function that was determined by the estimated glomerular filtration rate (eGFR) and be on medication for comorbidities. Patients with incomplete records were excluded from the study. Sample sizes were determined by applying a power analysis in G*Power, which used an alpha of 0.05, a power of 0.80 and a medium effect size of 0.4. The ratio of cases and controls was 1:2, and participants were selected by random sampling, matched by eGFR. Estimated glomerular filtration rate was stratified by the Kidney Disease Outcomes Quality Initiative (KDOQI) classification. Data were anonymised with reference numbers. Variables analysed included the following:

- age (years)
- sex
- HIV status
- type of DM
- duration of DM (years)
- duration of HIV (years)
- duration of ART (years)
- type of ART
- eGFR (mL/min/1.73m^2^)
- levels of serum sodium, potassium, corrected calcium and phosphate (mmol/L)
- levels of glycated haemoglobin (HbA1c) (%).

The following serum electrolyte reference ranges, as per the National Health Laboratory Services (NHLS), were utilised:

- sodium: 136 mmol/L – 145 mmol/L
- potassium: 3.5 mmol/L – 5.1 mmol/L
- calcium: 2.20 mmol/L – 2.55 mmol/L
- phosphate: 0.78 mmol/L – 1.42 mmol/L

Any electrolyte values below the lower limit of normal were considered hypo-electrolyte abnormalities, whilst those above the upper limit of normal were considered hyper-electrolyte abnormalities. Corrected serum calcium levels were utilised and calculated as follows: measured total calcium (mmol/L) + 0.02 (40 [g/L] – serum albumin [g/L]). The 2017 Society for Endocrinology, Metabolism and Diabetes of South Africa (SEMDSA) guidelines advocate for an HbA1c ≤ 7% to prevent micro- and macro-vascular complications. Therefore, in this study, adequate glycaemic control was defined as HbA1c ≤ 7%. Estimated glomerular filtration rate was calculated by using the Modification of Diet in Renal Disease (MDRD) formula. Serum electrolytes, eGFR and HbA1c were measured by using the Siemens Dimension^®^ analyser.

### Statistical analysis

Data were captured by using Microsoft Excel, version 2016 (Microsoft, USA). Statistical analyses were conducted by using Statistical Analysis Software (SAS), version 9.4 (SAS Institute Inc., Cary, NC, USA). Continuous variables were expressed as medians with interquartile ranges (IQRs). Categorical variables were expressed as frequencies and percentages. Continuous variables were compared by using the Wilcoxon rank-sum test as data were asymmetrically distributed. Categorical variables were compared by using either the Chi-square test or Fisher’s exact test if there were less than five observations in any cell. Pearson’s correlation coefficient assessed the correlation between HbA1c and electrolytes. Multinomial logistic regression assessed factors associated with electrolyte abnormalities. Linear regression analyses assessed the effect of measured covariates on electrolytes. Multivariable models were stratified by HIV status and were adjusted for gender, age, use of TDF, type of DM, duration of DM, duration of HIV, HbA1c and eGFR. A two-tailed value of *p* < 0.05 was considered to indicate statistical significance.

### Ethical consideration

Ethical approval to conduct the study was obtained from the Biomedical Research and Ethics Committee (BREC) of the University of KwaZulu-Natal (reference number BE576/18). Permission was obtained from Edendale hospital to utilise the DM clinic datasheet for data collection.

## Results

### Descriptive data

Ninety-six black African PLWH/DM (cases) and 192 black African HIV-uninfected patients with DM (controls) were reviewed. People living with HIV and DM were significantly younger than HIV-uninfected patients with DM (median [IQR]: 46.5 [39–53.5] years vs. 56 [47–64] years; *p* < 0.001) (Table 1). HIV-uninfected patients had a significantly longer duration of DM compared with PLWH/DM (median [IQR]: 7 [3–13] years vs. 5 [2–9] years; *p* = 0.006). People living with HIV and DM had a median (IQR) duration of HIV of 7 (3–10) years and 86 (89.58%) patients were on ART, which included 65 (67.7%) patients on TDF (Table 1). Eighty-four (87.5%) PLWH/DM and 172 (89.58%) HIV-uninfected patients had type 2 DM. Seventy-three (76.0%) PLWH/DM and 153 (79.7%) HIV-uninfected patients with DM had an HbA1c > 7%, which was considered uncontrolled. The median (IQR) HbA1c amongst PLWH/DM and HIV-uninfected patients with DM was 9.45% (7.1% – 11.45%) and 9.70% (7.35% – 11.5%), respectively (*p* = 0.836). An eGFR less than 60 mL/min/1.73m^2^ was present in 12 (12.5%) PLWH/DM and 24 (12.5%) HIV-uninfected patients with DM, respectively (Table1).

**TABLE 1:** Demographic and clinical characteristics of people living with human immunodeficiency virus and diabetes mellitus, and human immunodeficiency virus-uninfected patients with diabetes mellitus.

Parameter	PLWH/DM (*n* = 96)			HIV-uninfected patients with DM (*n* = 192)			*p*
	*n*	%	IQR	*n*	%	IQR	
Age (years), median	46.5	-	39–53.5	56	-	47–64	< 0.001
**Gender**							1.000
Female	66	68.75	-	132	68.75	-	
Male	30	31.25	-	60	31.25	-	
Duration of DM (years), median	5	-	2–9	7	-	3–13	0.006
Duration of HIV (years), median	7	-	3–10	-	-	-	-
Duration on ART (years), median	6	-	2–9	-	-	-	-
Sodium (mmol/L), median	140	-	136.5–142	140	-	138–142	0.072
Potassium (mmol/L), median	4.35	-	3.90–4.70	4.3	-	3.90–4.70	0.903
Calcium (mmol/L), median	2.24	-	2.18–2.30	2.29	-	2.20–2.36	0.001
Phosphate (mmol/L), median	1.03	-	0.91–1.21	1.08	-	0.93–1.23	0.406
HbA1c (%), median	9.45	-	7.1–11.5	9.7	-	7.35–11.50	0.836
HbA1c > 7%	73	76.04	-	153	79.69	-	0.543
eGFR < 60 mL/min/1.73m^2^	12	12.5	-	24	12.5	-	1.000
**Diabetes type**							0.691
Type 1	12	12.5	-	20	10.41	-	
Type 2	84	87.5	-	172	89.58	-	
**Sodium**							0.457
Hyponatraemia	18	18.75	-	26	13.54	-	
Hypernatraemia	2	2.08	-	7	3.65	-	
**Potassium**							0.851
Hypokalaemia	6	6.25	-	15	7.81	-	
Hyperkalaemia	8	8.33	-	19	9.90	-	
**Calcium**							0.292
Hypocalcaemia	30	31.25	-	44	22.91	-	
Hypercalcaemia	1	1.04	-	3	1.56	-	
**Phosphate**							0.959
Hypophosphataemia	5	5.21	-	12	6.25	-	
Hyperphosphataemia	6	6.25	-	12	6.25	-	
On ART	86	89.58	-	-	-	-	-
On TDF ART	65	67.71	-	-	-	-	-

*Source:* Edendale Hospital diabetes clinic datasheet

PLWH/DM, people living with human immunodeficiency virus and diabetes mellitus; HIV, human immunodeficiency virus; DM, diabetes mellitus; IQR, interquartile range; HbA1c, glycated haemoglobin; eGFR, estimated glomerular filtration rate; ART, antiretroviral therapy; TDF, tenofovir.

### Analysis of electrolytes

#### Sodium

Hyponatraemia was the second most frequent electrolyte abnormality, which occurred in 18 (18.75%) PLWH/DM and 26 (13.54%) HIV-uninfected patients with DM (Table 1). Serum sodium was the only electrolyte significantly negatively correlated with HbA1c in both PLWH/DM (*r* = −0.34; *p* = 0.001) and HIV-uninfected patients with DM (*r* = −0.28; *p* < 0.001) (Table 2). Adjusted multinomial logistic regression analysis amongst PLWH/DM suggests that for every per cent increase in HbA1c, the odds of hyponatraemia significantly increased by 55% (odds ratio [OR]: 1.55; 95% confidence interval [CI]: 1.19–2.02; *p* = 0.003), whilst in HIV-uninfected patients with DM the odds of hyponatraemia significantly increased by 26% (OR: 1.26; 95% CI: 1.04–1.54; *p* = 0.009) (Table 3). Multivariate linear regression showed significant associations between serum sodium and HbA1c. Amongst PLWH/DM, for every per cent increase in HbA1c, serum sodium decreased by 0.51 mmol/L (β = −0.51; *p* = 0.004), and for every per cent increase in HbA1c amongst HIV-uninfected patients with DM, serum sodium decreased by 0.45 mmol/L (β = −0.45; *p* < 0.001) (Table 4).

**TABLE 2:** Correlation of electrolytes and glycated haemoglobin in people living with human immunodeficiency virus and diabetes mellitus, and human immunodeficiency virus-uninfected patients with diabetes mellitus.

Electrolyte	PLWH/DM		HIV-uninfected patients with DM	
	*r*	*p*	*r*	*p*
Sodium	−0.340	0.001	−0.28	< 0.001
Potassium	0.090	0.397	−0.08	0.283
Calcium	0.764	0.475	0.05	0.475
Phosphate	0.110	0.302	−0.05	0.473

*Source:* Edendale Hospital diabetes clinic datasheet

*r* = Pearson’s correlation coefficient.

PLWH/DM, people living with HIV and diabetes mellitus; HIV, human immunodeficiency virus; DM, diabetes mellitus.

**TABLE 3:** Adjusted and un-adjusted odds ratio estimates for the associations of electrolyte abnormalities with glycated haemoglobin in people living with human immunodeficiency virus and diabetes mellitus, and human immunodeficiency virus-uninfected patients with diabetes mellitus.

Parameter	PLWH/DM						HIV-uninfected patients with DM					
	Univariate			Multivariate			Univariate			Multivariate		
	OR	95% CI	*p*	aOR	95% CI	*p*	OR	95% CI	*p*	aOR	95% CI	*p*
Hyponatraemia	1.39	1.13–1.71	0.007	1.55	1.19–2.02	0.003	1.22	1.01–1.46	0.009	1.26	1.04–0.54	0.009
Hypernatraemia	0.99	0.57–1.73	-	0.16	0.00–10.50	-	0.64	0.44–0.95	-	0.67	0.44–1.02	-
Hypokalaemia	1.10	0.84–1.45	0.565	1.20	0.72–1.99	0.736	0.98	0.79–1.22	0.215	0.99	0.80–1.23	0.089
Hyperkalaemia	1.11	0.88–1.42	-	1.05	0.82–1.35	-	0.83	0.68–1.02	-	0.75	0.58–0.97	-
Hypocalcaemia	0.91	0.78–1.07	0.467	0.89	0.75–1.06	0.426	0.89	0.77–1.03	0.226	0.91	0.79–1.05	0.321
Hypercalcaemia	0.76	0.32–1.84	-	0.03	0.00–1.27884E12	-	0.82	0.51–1.34	-	0.78	0.43–1.42	-
Hypophosphataemia	0.80	0.54–1.20	0.017	0.76	0.48–1.22	0.078	1.01	0.79–1.29	0.199	1.01	0.79–1.29	0.250
Hyperphosphataemia	1.53	1.12–2.11	-	1.44	1.00–2.07	-	0.79	0.61–1.02	-	0.79	0.60–1.04	-

*Source:* Edendale Hospital diabetes clinic datasheet

OR, odds ratio; aOR, adjusted odds ratio; CI, confidence interval; DM, diabetes mellitus; HbA1c, glycated haemoglobin; PLWH/DM, people living with HIV and diabetes mellitus; HIV, human immunodeficiency virus.

**TABLE 4:** Linear regression analysis for the associations of electrolytes with clinical factors in people living with human immunodeficiency virus and diabetes mellitus, and human immunodeficiency virus-uninfected patients with diabetes mellitus.

Parameter	PLWH/DM				HIV-uninfected patients with DM			
	Univariate		Multivariate		Univariate		Multivariate	
	Estimate	*p*	Estimate	*p*	Estimate	*p*	Estimate	*p*
**Age**								
Sodium	0.05	0.346	0.09	0.145	−0.03	0.111	−0.02	0.336
Potassium	−0.01	0.905	0.01	0.999	0.04	0.281	−0.04	0.360
Calcium	−0.01	0.526	−0.01	0.663	−0.01	0.018	−0.01	0.031
Phosphate	−0.01	0.167	−0.01	0.033	−0.01	0.939	−0.01	0.226
**HbA1c**								
Sodium	−0.59	0.001	−0.51	0.004	−0.43	< 0.001	−0.45	< 0.001
Potassium	0.02	0.397	0.02	0.512	−0.02	0.283	−0.02	0.290
Calcium	0.01	0.764	0.01	0.681	0.02	0.475	0.01	0.637
Phosphate	0.01	0.302	0.01	0.740	−0.01	0.473	−0.01	0.296
**Duration of DM**								
Sodium	−0.02	0.820	0.02	0.850	−0.07	0.047	−0.02	0.507
Potassium	−0.02	0.181	−0.04	0.018	0.02	0.004	0.01	0.042
Calcium	0.01	0.547	0.01	0.370	0.01	0.900	0.01	0.187
Phosphate	0.01	0.475	−0.01	0.834	0.03	0.096	0.01	0.251
**Duration of HIV**								
Sodium	0.14	0.195	0.08	0.473	-	-	-	-
Potassium	−0.02	0.334	−0.02	0.345	-	-	-	-
Calcium	0.01	0.653	0.01	0.268	-	-	-	-
Phosphate	0.01	0.710	0.01	0.255	-	-	-	-
**eGFR: Abnormal (ref: ≥ 60 mL/min/1.73m^2^)**								
Sodium	2.96	0.054	2.48	0.111	1.83	0.024	1.75	0.037
Potassium	−0.56	0.017	−0.68	0.007	0.04	0.711	−0.49	0.001
Calcium	0.02	0.514	−0.04	0.164	0.02	0.436	0.02	0.495
Phosphate	−0.21	0.003	−0.25	0.001	−0.07	0.139	−0.08	0.077
**Gender: Female (ref: male)**								
Sodium	1.57	0.155	1.69	0.116	−0.73	0.208	−0.58	0.318
Potassium	0.19	0.252	0.26	0.123	−0.54	0.000	0.07	0.504
Calcium	−0.03	0.219	0.02	0.546	−0.02	0.231	−0.03	0.231
Phosphate	0.10	0.059	0.09	0.072	0.08	0.012	0.09	0.006
**DM Type: Type 1 (ref: Type 2)**								
Sodium	1.13	0.465	2.88	0.114	0.41	0.640	−0.08	0.943
Potassium	−0.04	0.877	0.04	0.888	−0.10	0.501	−0.12	0.513
Calcium	0.03	0.455	0.01	0.774	0.04	0.248	−0.02	0.517
Phosphate	0.03	0.681	−0.03	0.682	−0.01	0.997	−0.01	0.925
**Type of ART: TDF (ref: not on TDF)**								
Sodium	−0.58	0.597	0.50	0.629	-	-	-	-
Potassium	−0.07	0.695	−0.08	0.613	-	-	-	-
Calcium	−0.04	0.095	−0.05	0.078	-	-	-	-
Phosphate	−0.05	0.337	−0.07	0.169	-	-	-	-

DM, diabetes mellitus; HbA1c, glycated haemoglobin; eGFR, estimated glomerular filtration rate; ART, antiretroviral therapy; TDF, tenofovir; PLWH/DM, people living with HIV and diabetes mellitus; HIV, human immunodeficiency virus.

#### Potassium

The duration of HIV was not significantly associated with hypokalaemia on adjusted multinomial logistic regression analysis (OR: 0.85; 95% CI: 0.59–1.23; *p* = 0.645). Furthermore, the odds of hypokalaemia in PLWH using TDF compared with non-TDF-based ART were not significant (OR: 0.87; 95% CI: 0.06–13.12; *p* = 0.766). Adjusted multinomial logistic regression determined that the duration of DM was significantly associated with potassium abnormalities. In PLWH/DM, for every year increase in the duration of DM, the odds of hypokalaemia increased by 97% (OR: 1.97; 95% CI: 1.13–3.43; *p* = 0.025). However, in HIV-uninfected patients with DM, the odds of hyperkalaemia increased by 10% (OR: 1.10; 95% CI: 1.02–1.19; *p* = 0.048). Multivariate linear regression also showed significant associations between serum potassium levels and the duration of DM. For every year increase in the duration of DM, serum potassium decreased by 0.04 mmol/L amongst PLWH/DM (β = −0.04; *p* = 0.018) and increased by 0.01 mmol/L amongst HIV-uninfected patients with DM (β = 0.01; *p* = 0.042) (Table 4).

#### Calcium

Serum-corrected calcium was the only electrolyte with median (IQR) levels significantly lower in PLWH/DM compared with HIV-uninfected patients with DM (2.24 [2.18–2.30] mmol/L vs. 2.29 [2.20–2.36] mmol/L; *p* = 0.001). Furthermore, the most frequent electrolyte abnormality in PLWH/DM and HIV-uninfected patients with DM was hypocalcaemia (31.25% vs. 22.91%) (Table 1). Adjusted multinomial logistic regression in PLWH/DM and HIV-uninfected patients with DM found no factors significantly associated with hypocalcaemia or hypercalcaemia. However, multivariate linear regression analysis in HIV-uninfected patients with DM showed that for every year increase in age, serum calcium decreased by 0.01 mmol/L (β = −0.01; *p* = 0.031) (Table 4).

#### Phosphate

Adjusted multinomial logistic regression in PLWH/DM and HIV-uninfected patients with DM found no factors significantly associated with hypophosphataemia or hyperphosphataemia. Notably, the use of TDF was not significantly associated with hypophosphataemia in PLWH/DM (OR: 1.69; 95% CI: 0.20–14.12; *p* = 0.800). Multivariate linear regression in PLWH/DM determined that for every year increase in age, serum phosphate decreased by 0.01 mmol/L (β = −0.01; *p* = 0.033). Moreover, on average, serum phosphate was 0.09 mmol/L higher in women than in men amongst HIV-uninfected patients with DM, and all other variables were constant (β = 0.09; *p* = 0.006) (Table 4).

## Discussion

### Sodium

Serum sodium abnormalities in DM vary depending on the degree of water and sodium change.^9^ Serum glucose is an osmotically active substance; therefore, hyponatraemia in DM is mostly attributed to hyperglycaemia-induced hyper-osmolality, resulting in a dilutional effect or osmotic diuresis with hypovolemic hyponatraemia.^9^ Hypernatraemia may occur if water loss exceeds sodium loss.^9^ Our study identified serum sodium to be the only electrolyte significantly associated with HbA1c levels in both PLWH/DM and HIV-uninfected patients with DM. Furthermore, elevated HbA1c levels significantly increased the odds of hyponatraemia, with the odds being greater in PLWH/DM compared with their HIV-uninfected counterparts. Although pseudo-hyponatraemia in DM is common, hyponatraemia could be utilised as a marker of impaired DM control. Our finding regarding the association between HbA1c and serum sodium levels is comparable with that of a study conducted in India, which determined that mean (standard deviation [s.d.]) serum sodium levels were significantly lower in patients with DM compared with non-DM controls (127.92 [0.45] mmol/L vs. 135.82 [0.34] mmol/L; *p* = 0.0001) and that HbA1c was significantly inversely correlated with serum sodium levels (*r* = 0.640; *p* = 0.0001).^7^ However, no regression analysis was performed in the study conducted in India.^7^ Other causes of hyponatraemia in DM include side effects of drugs such as diuretics, diabetic nephropathy and the syndrome of inappropriate antidiuretic hormone secretion (SIADH).^9^

Our study demonstrated that PLWH/DM had a higher frequency of hyponatraemia compared with HIV-uninfected patients with DM (18.75% vs. 13.4%). The increased frequency of hyponatraemia in PLWH/DM could be attributed to the additive effect of HIV on sodium homeostasis. Hyponatraemia is a common electrolyte disorder in PLWH and a possible marker of HIV severity, as patients with hyponatraemia have significantly lower CD4 counts, higher viral loads and an increased prevalence of acquired immunodeficiency syndrome (AIDS).^16^ In PLWH, the main causes of hyponatraemia include opportunistic infections which predispose to SIADH, adrenal insufficiency, diarrhoea and vomiting.^17^ Furthermore, dysfunction of the thick ascending limb of the loop of Henle secondary to HIV and inflammation results in impaired free water clearance and dilutional hyponatraemia.^8,18^

People living with HIV and DM may be at a higher risk of hyponatraemia because of contributory factors from both HIV and DM. Furthermore, hyponatraemia in the black African population may indicate a greater degree of sodium imbalance compared with other ethnic groups as the black population physiologically have increased sodium retention with lower plasma renin and aldosterone levels.^19,20^

### Potassium

Hyperkalaemia is common in DM.^7,21,22^ Conversely, studies conducted in Nigeria and Saudi Arabia found a predominant hypokalaemia and no significant association between serum potassium levels and glycaemic control, respectively.^23,24^ Similarly, our study found no significant association between HbA1c and serum potassium levels. Common causes of hyperkalaemia in DM and HIV include hyporeninaemic hypo-aldosteronism, acidosis, renal impairment and drugs such as angiotensin-converting enzyme (ACE) inhibitors, potassium-sparing diuretics and beta-blockers^8,9^ Hypokalaemia in DM is frequently caused by insulin administration, malabsorption, osmotic diuresis and hypomagnesaemia.^9^ In PLWH, hypokalaemia is commonly caused by vomiting, diarrhoea and proximal tubular dysfunction secondary to TDF.^25,26,27^ However, in our study, TDF was not significantly associated with hypokalaemia. This could be attributed to our study having relatively young PLWH that were on ART for a median duration of 6 years and only 12.5% of PLWH having an eGFR < 60ml/min/1.73m^2^, which may reduce the risk of TDF induced nephrotoxicity.

Notably, our study determined that for every unit increase in DM duration, the odds of hypokalaemia significantly increased by 97% in PLWH/DM. This could be attributed to patients with comorbid HIV and DM developing a greater degree of insulin resistance, as both conditions progress,^28^ and therefore require higher doses of insulin to achieve glycaemic control with a propensity for hypokalaemia. This possible contributory factor needs to be explored further as our study did not evaluate the use of insulin. In HIV-uninfected patients with DM, the likelihood of hyperkalaemia significantly increased by 10% for every unit increase in DM duration. This could be attributed to dysautonomia in long-standing DM which impairs the conversion of prorenin to renin and predisposes to hyporeninaemic hypo-aldosteronism and associated hyperkalaemia.^29^

### Calcium

Calcium homeostasis is strongly regulated by parathyroid hormone and vitamin D. Factors contributing to hypocalcaemia in HIV and DM include vitamin D deficiency, hypoparathyroidism and hypomagnesaemia.^9,30^ Our study identified hypocalcaemia as the most common electrolyte abnormality in both PLWH/DM and HIV-uninfected patients with DM. Furthermore, serum calcium was the only electrolyte with median levels significantly lower in PLWH/DM compared with HIV-uninfected patients with DM. Similarly, Keuhn et al. identified mean serum calcium levels to be significantly lower in HIV-infected patients compared with controls (*p* < 0.0001), irrespective of serum albumin levels.^30^ Hypocalcaemia is also common in patients with DM, with a study in Sudan showing significantly lower mean serum calcium levels in patients with DM compared with controls (*p* < 0.05).^31^ Furthermore, vitamin D deficiency in the elderly is common despite consistent vitamin D intake and may predispose to hypocalcaemia.^32^ Notably, our study demonstrated a significant inverse association between serum calcium and age in HIV-uninfected patients with DM. A significant association between serum calcium and age may have not been detected in PLWH/DM as they were significantly younger. The degree of sunlight exposure was not documented in this study. Although PLWH/DM were significantly younger than HIV-uninfected patients with DM, clinically this difference in age should not usually result in a greater proportion of HIV-uninfected patients being housebound. Therefore, the significant inverse association between serum calcium levels and age in HIV-uninfected patients may be influenced by factors besides sun exposure.

The mechanism of vitamin D deficiency in HIV is multifactorial and involves the inhibitory effect of pro-inflammatory cytokines that reduces renal 1-α hydroxylation of vitamin D and the consumption of vitamin D by macrophages and lymphocytes.^33^ Furthermore, vitamin D has a significant immunomodulatory role, and deficiencies in PLWH are associated with lower CD4 cell counts, higher viral loads, HIV progression and an increased risk of opportunistic infections.^34^ Moreover, studies have suggested that vitamin D deficiency and low calcium levels result in impaired insulin synthesis and secretion with subsequent glucose intolerance and insulin resistance.^15^ The European AIDS Clinical Society (EACS) has vitamin D supplementation recommendations and reports that the prevalence of low vitamin D levels was up to 80% in HIV cohorts and was associated with an increased risk of osteoporosis, type 2 DM, mortality and AIDS events.^35^ This is in contrast to South African HIV and DM guidelines, which do not have vitamin D deficiency recommendations despite our susceptible population.^36,37^

Consequently, the presence of comorbid HIV and DM in the black African population potentially increases the risk of vitamin D deficiency and hypocalcaemia, which might negatively impact HIV and DM control. The role of vitamin D in the pathogenesis and control of HIV and DM and the effect of vitamin D supplementation need to be further explored, particularly in the black African population.

### Phosphate

The risk of TDF-induced nephrotoxicity with isolated hypophosphataemia, proximal tubular dysfunction or Fanconi syndrome increases in the presence of renal impairment.^38^ This is of particular concern in patients with comorbid HIV and DM, advancing age, lower CD4 cell counts and elevated baseline creatinine levels.^38^ Our study did not find TDF to be significantly associated with hypophosphataemia or serum phosphate levels in PLWH/DM. This could be attributed to the fact that our study had only 12.5% of PLWH/DM with an eGFR <60 mL/min/1.73m^2^, patients were using ART for a median duration of only 6 years and PLWH/DM were relatively young. A study by Day et al. observed the frequency of hypophosphataemia in TDF recipients to be higher than non-TDF ART recipients (31% vs. 22%). However, no independent association was found between TDF use and the frequency or severity of hypophosphataemia.^39^ The recognition that hypophosphataemia in PLWH on TDF is multifactorial must be considered to avoid unnecessary TDF cessation in a resource-limited setting.

### Current guidelines

This study determined that electrolyte abnormalities in black African PLWH/DM are common, with hypocalcaemia and hyponatraemia being the most frequent electrolyte abnormalities. However, the current SEMDSA guidelines only recommend that serum potassium needs to be measured at diagnosis and monitored annually.^37^ Furthermore, South African HIV guidelines only recommend the monitoring of serum potassium and phosphate in high-risk patients and patients with features of tubular wasting.^36^

## Limitations

The limitations of this study included CD4 count and viral load not being documented for a majority of patients as management and monitoring of HIV occurs at designated HIV clinics. Therefore, the association between HIV control and electrolyte abnormalities could not be determined. Possible TDF-induced proximal tubular dysfunction and electrolyte loss were not assessed with urine electrolytes as they were not routinely performed in the DM clinic. Patients in this study could have been on medication or could have comorbidities which may affect electrolytes. However, by including these patients the study was more representable and reproducible as the majority of patients suffering from DM were usually part of a metabolic syndrome which requires chronic treatment to improve outcomes. In addition, the use of oral antidiabetic medication or insulin and the respective doses were not included. This could have been used to compare the DM treatment requirements in PLWH/DM and HIV-uninfected patients as both groups had similar HbA1c levels. Lastly, because of the retrospective nature of the study, causality could not be determined. However, this is a newly explored topic, and this study is useful in providing preliminary data for future prospective studies.

## Conclusion

Serum electrolyte abnormalities in black African PLWH/DM are common. Hypocalcaemia and hyponatraemia were the most frequent electrolyte abnormalities and occurred more frequently in PLWH/DM compared with HIV-uninfected patients with DM. Serum calcium levels were significantly lower in black African PLWH/DM compared with HIV-uninfected patients with DM. Importantly, hyponatraemia is a potential marker of impaired glycaemic control as elevated HbA1c levels significantly increased the odds of hyponatraemia in both groups; however, the odds were greater in PLWH/DM. Ultimately, black African PLWH/DM are highly vulnerable to electrolyte abnormalities because of multifactorial pathophysiological factors. Further large prospective studies regarding electrolyte abnormalities in black African PLWH/DM will assist in identifying contributory factors and implementing tailored guidelines that could facilitate prevention, earlier detection, closer monitoring and appropriate intervention to reduce associated adverse effects in this high-risk population, particularly in the South African context.

## References

[1] International Diabetes Federation IDF Diabetes Atlas, 8th ed. [homepage on the Internet]. Brussels: International Diabetes Federation; 2017 [cited 2019 Mar 03]. Available from: http://www.diabetesatlas.org

[2] Statistics South Africa Media release: Mortality and causes of death, 2015 [hompage on the Internet]. Statistics South Africa [cited 2019 Mar 03]. Available from: http://www.statssa.gov.za/?p=9604

[3] UNAIDS South Africa overview [homepage on the Internet]. [cited 2019 Mar 03]. Available from: https://www.unaids.org/en/regionscountries/countries/southafrica

[4] Statistics South Africa Mid-year population estimates 2017 [homepage on the Internet]. [cited 2019 Mar 03]. Available from: http://www.statssa.gov.za/publications/P0302/P03022017.pdf

[5] Samad F, Harris M, Puskas C, et al Incidence of diabetes mellitus and factors associated with its development in HIV-positive patients over the age of 50. BMJ Open Diabetes Res Care. 2017;5(1):e000457 10.1136/bmjdrc-2017-000457 PMC5717418 29225896

[6] Hernandez-Romieu A, Garg S, Rosenberg E, Thompson-Paul A, Skarbinski J Is diabetes prevalence higher among HIV-infected individuals compared with the general population? Evidence from MMP and NHANES 2009–2010. BMJ Open Diabetes Res Care. 2017;5(1):e000304 10.1136/bmjdrc-2016-000304 PMC5293823 28191320

[7] Rajagopal L, Ganesan V, Abdullah S, et al Exploring the interrelationship between electrolytes, anemia, and glycosylated hemoglobin (HbA1c) levels in type 2 diabetics. Asian J Pharm Clin Res. 2018;11(1):251 10.22159/ajpcr.2017.v11i1.22533

[8] Musso C, Belloso W, Glassock R Water, electrolytes, and acid-base alterations in human immunodeficiency virus infected patients. World J Nephrol. 2016;5(1):33–42. 10.5527/wjn.v5.i1.33 26788462 PMC4707166

[9] Liamis G, Liberopoulos E, Barkas F, Elisaf M Diabetes mellitus and electrolyte disorders. World J Clin Cases: WJCC. 2014;2(10):488–496. 10.12998/wjcc.v2.i10.488 25325058 PMC4198400

[10] Naicker S, Rahmania S, Kopp J HIV and chronic kidney disease. Clin Nephrol. 2015;83(Suppl 1):32–38. 10.5414/cnp83s032 25725239 PMC4536633

[11] Labarga P, Barreiro P, Martin-Carbonero L, et al Kidney tubular abnormalities in the absence of impaired glomerular function in HIV patients treated with tenofovir. AIDS. 2009;23(6):689–696. 10.1097/qad.0b013e3283262a6 19262355

[12] Brancati F, Kao W, Folsom A, et el Incident type 2 diabetes mellitus in African American and white adults: The atherosclerosis risk in communities study. JAMA. 2000;283(17):2253–2259. 10.1001/jama.283.17.2253 10807384

[13] Alvarez J, Bush N, Choquette S, et al Vitamin D intake is associated with insulin sensitivity in African American, but not European American, women. Nutr Metab. 2010;7(1):28 10.1186/1743-7075-7-28 PMC2868016 20398267

[14] Chatterjee R, Yeh H-C, Shafi T, et al Serum potassium and the racial disparity in diabetes risk: The Atherosclerosis Risk in Communities (ARIC) study. Am J Clin Nutr. 2011;93(5):1087–1091. 10.3945/ajcn.110.007286 21367942 PMC3076658

[15] Davis S Vitamin D deficiency and type 2 diabetes in African Americans: The common denominators. Diabetes Spectrum. 2011;24(3):148 10.2337/diaspect.24.3.148

[16] Braconnier P, Delforge M, Garjau M, Wissing K, De Wit S Hyponatremia is a marker of disease severity in HIV-infected patients: A retrospective cohort study. BMC Infect Dis. 2017;17(1):98 10.1186/s12879-017-2191-5 28122494 PMC5267411

[17] Shu Z, Tian Z, Chen J, et al HIV/AIDS-related hyponatremia: An old but still serious problem. Renal Failure. 2018;40(1):68–74. 10.1080/0886022x.2017.1419975 29299949 PMC6014325

[18] Belloso W, de Paz Sierra M, Navarro M, Sanchez M, Perelsztein A, Musso C Impaired urine dilution capability in HIV stable patients. Int J Nephrol. 2014;2014:1–8. 10.1155/2014/381985 PMC3988737 24800076

[19] Sagnella G Why is plasma renin activity lower in populations of African origin? J Hum Hypertens. 2001;15(1):17–25. 10.1038/sj.jhh.1001127 11223998

[20] Rayner B, Myers J, Opie L, Trinder Y, Davidson J Screening for primary aldosteronism – Normal ranges for aldosterone and renin in three South African population groups. S Afr Med J. 2001;91(7):594–599. 10.1177/1470320312463833 11544978

[21] Anago E, Medehouenou T, Akpovi CD, Tchehouenou H Electrolyte disturbances in diabetic patients in Cotonou, Benin. Int J Res Med Sci. 2016;4:5430–5435. 10.18203/2320-6012.ijrms20164223

[22] McNair P, Madsbad S, Christiansen C, Christensen M, Transbol I Hyponatremia and hyperkalemia in relation to hyperglycemia in insulin-treated diabetic out-patients. Clin Chim Acta. 1982;120(2):243–250. 10.1016/0009-8981(82)90161-9 7039873

[23] E. Ugwuja N A comparative study of serum electrolytes, total protein, calcium and phosphate among diabetic and HIV/AIDS patients in Abakaliki, Southeastern, Nigeria. Internet J Lab Med. 2007;2(1):1–5. 10.5580/15e5

[24] Al-Rubeaan K, Siddiqui K, Abu Risheh K, et al Correlation between serum electrolytes and fasting glucose and Hba1c in Saudi diabetic patients. Biol Trace Element Res. 2011;144(1–3):463–468. 10.1007/s12011-011-9144-4 21818670

[25] Eshiet E, Okogun G Plasma urea and electrolytes profile in different stages of hiv infection in Ekpoma, Nigeria. Afr J Cell Pathol. 2015;4(1):1–5. 10.5897/ajcpath15.001

[26] Narayanan Ramesh V To study the renal and electrolyte disturbances in HIV infected patients. IOSR Jof Dent Med Sci. 2017;16(8):91–94.

[27] Emejulu A, Onwuliri V, Ojiako O Electrolyte abnormalities and renal impairment in asymptomatic HIV-infected patients in Owerri, South Eastern Nigeria. Aust J Basic Appl Sci. 2011;5(3):257–260.

[28] Kalra S, Kalra B, Agrawal N, Unnikrishnan A Understanding diabetes in patients with HIV/AIDS. Diabetol Metab Syndr. 2011;3(1):2 10.1186/-1758-5996-3-2 21232158 PMC3025836

[29] McFarlane S, Sowers J Aldosterone function in diabetes mellitus: Effects on cardiovascular and renal disease. J Clin Endocrinol Metab. 2003;88(2):516–523. 10.1210/jc.2002-021443 12574172

[30] Kuehn E, Anders H, Bogner J, Obermaier J, Goebel F, Schlondorff D Hypocalcaemia in HIV infection and AIDS. J Intern Med. 1999;245(1):69–73. 10.1046/j.1365-2796.1999.00407.x 10095819

[31] Hassan S, Elsheikh W, Rahman N, ElBagir N Serum calcium levels in correlation with glycated hemoglobin in type 2 diabetic sudanese patients. Adv Diabetes Metab. 2016;4(4):59–64.

[32] Linnebur S, Vondracek S, Vande Griend J, Ruscin J, McDermott M Prevalence of vitamin D insufficiency in elderly ambulatory outpatients in Denver, Colorado. Am J Geriatr Pharmacother. 2007;5(1):1–8. 10.1016/j.amjopharm.2007.03.005 17608242

[33] Villamor E A potential role for vitamin D on HIV infection? Nutr Rev. 2006;64(5):226–233. 10.1111/j.1753-4887.2006.tb00205.x 16770943

[34] Alvarez N, Aguilar-Jimenez W, Rugeles M The potential protective role of vitamin D supplementation on HIV-1 infection. Front Immunol. 2019;10 10.3389/fimmu.2019.02291 31611877 PMC6773828

[35] European AIDS Clinical Society guidelines 2018 [homepage on the Internet]; version 9.1:49–51. [cited 2019 Mar 03]. Available from: www.eacsociety.org/files/2018_guidelines-9.1-english.pdf

[36] Meintjes G, Moorhouse M, Carmona S, et al Adult antiretroviral therapy guidelines 2017. S Afr J HIV Med. 2017;18(1):a776 10.4102/sajhivmed.v18i1.776 PMC5843236 29568644

[37] SEMDSA 2017 guidelines for the management of type 2 diabetes mellitus SEMDSA type 2 Diabetes Guidelines Expert Committee. JEMSDA [serial online] 2017 [cited 2019 Mar 03];22(1):1–196. Available from: https://www.semdsa.org.za/images/647-4385-1-pb.pdf

[38] Tourret J, Deray G, Isnard-Bagnis C Tenofovir effect on the kidneys of HIV-infected patients: A double-edged sword? J Am Soc Nephrol. 2013;24(10):1519–1527. 10.1681/asn.2012080857 24052632 PMC3785270

[39] Day S, Leake Date H, Bannister A, Hankins M, Fisher M Serum hypophosphatemia in tenofovir disoproxil fumarate recipients is multifactorial in origin, questioning the utility of its monitoring in clinical practice. JAIDS J Acquir Immune Defic Syndr. 2005;38(3):301–304. 15735448

